# Evaluation of the Potential Release Tendency of Metals and Metalloids from the Estuarine Sediments: Case Study of Raša Bay

**DOI:** 10.3390/molecules26216656

**Published:** 2021-11-03

**Authors:** Željka Fiket, Marija Petrović, Gordana Medunić, Tatjana Ivošević, Tomislav Fiket, Lizzy Zhang Xu, Yan Wang, Shiming Ding

**Affiliations:** 1 Divison for Marine and Environmental Research, Ruđer Bošković Institute, Bijenička Cesta 54, HR-10000 Zagreb, Croatia; petrovic@irb.hr; 2 Department of Geology, Faculty of Science, University of Zagreb, Horvatovac 95, HR-10000 Zagreb, Croatia; gordana.medunic@geol.pmf.hr; 3 Faculty of Maritimes Studies, University of Rijeka, Studenska 2, HR-51000 Rijeka, Croatia; tatjana.ivosevic14@gmail.com; 4 Department of Geophysics, Faculty of Science, University of Zagreb, Horvatovac 95, HR-10000 Zagreb, Croatia; tfiket@gfz.hr; 5 Nanjing Easysensor Environmental Technology Co., Ltd., Nanjing 210018, China; lizzyzhangxu@163.com (L.Z.X.); yuyan19881116@163.com (Y.W.); 6 State Key Laboratory of Lake Science and Environment, Nanjing Institute of Geography and Limnology, Chinese Academy of Sciences, Nanjing 210008, China; smding@niglas.ac.cn

**Keywords:** estuarine sediments, oxyanions, DGT, geochemical composition

## Abstract

Assessing the environmental quality of coastal systems is important not only for the management and protection of such areas, but also for improving the quality of water resources. Since sediment itself can often be a source of certain toxic elements, in addition to information on the distribution of metals in the water column and in the sediment itself, it is useful to determine the bioavailable forms of individual elements, particularly toxic ones. In this study, water and sediment geochemical data were supplemented with oxyanion mobility in sediments estimated by diffusion gradients in thin film (DGTs). The data obtained indicate that the chemical composition of the water in the Raša River estuary primarily reflects the high input of suspended sediment from the catchment, the mixing of freshwater and seawater, and to a lesser extent the effects of anthropogenic activities. Although sediment composition is primarily determined by geological and hydrodynamic conditions in the catchment, it also indicates moderate enrichment in Co, Cr, Mo and Ni. In contrast, the distribution of oxyanions in sediment pore water indicates the influence of sediment as a source of some elements in the bottom water; e.g., sediment contributes to 40% of the arsenic bottom water budget. The obtained depth profiles of the oxyanion distribution in the sediment pore water indicate an early onset of suboxic to anoxic conditions in Raša Bay, which is prone to rapid sedimentation. All this demonstrates the need to consider the bioavailable forms of elements when assessing environmental quality, as the lack of such information can lead to an incomplete assessment, especially in dynamic coastal systems such as estuaries.

## 1. Introduction

The Adriatic Sea is part of the Mediterranean Sea. Separated by the Italian Peninsula and the Balkan Peninsula, it extends from the Gulf of Venice southward to the Strait of Otranto. Most of its eastern coast belongs to Croatia. Despite minor polluted zones, the Adriatic Sea is a fairly clean sea. A comparison of concentrations of detergents, oils, phenols, heavy metals and other pollutants has shown that the input to the Adriatic Sea is the lowest compared to the Black Sea and the Baltic Sea. The most sensitive area, however, is the northwestern coast, where the polluted waters of the Po cause eutrophication and promote algal blooms [1]. The northeastern coast of the Adriatic (Istrian peninsula) is less affected, but the influences of the centuries-old tradition of Raša coal mining in this area cannot be overlooked. The entire region is marked by the remnants of coal mining and its decades-long use in nearby thermal power plants and factories. The negative impacts of Raša coal mining and its use in the local industrial sector are reflected in the elevated levels of various inorganic and organic pollutants in local watercourses, soils, sediments and biota [2,3]. Since near-shore marine systems are particularly vulnerable to pollution by numerous pollutants carried into them by rivers, the focus of this study is on the estuary of the Raša River, whose catchment area coincides with the area of the aforementioned activities.

The Raša River estuary is a region of intense deposition of fine-grained, mostly clayey particles derived from Eocene clastic flysch sediments, and represents a river-dominated disequilibrium estuary [4]. Most of the particles (90%) carried into the estuary by the Raša River are suspended solids, and at the head of the estuary this fine-grained material is flocculated and preferentially accumulates, creating a progradation delta [4]. 

Since the geochemistry of coal ash is largely different from that of natural rocks, soils and sediments [5], it is likely that the geochemistry of the affected natural system will change once the ash is released into the environment. This facilitates the use of geochemical methods, especially trace elements, to detect the possible presence of ash and the associated environmental contamination [6].

While water is a dynamic medium with variable composition, sediments can accumulate long-term persistent pollutants that have the potential for remobilization and biomagnification. They serve as reservoirs for various metals and metalloids, which can also become their resources in certain environments [7,8,9]. Indeed, changes in environmental conditions, e.g., redox potential, pH and/or oxygen balance, favour their release back into the aquatic system. In aquatic systems, bioaccumulative elements can reach high concentrations and have negative ecological effects. The mobility and availability of oxyanions in water and sediment is influenced by a number of properties such as pH, Fe/Mn oxyhydroxides, organic matter, and competition between oxyanions [10,11,12,13]. Diffusion gradients in thin film (DGT) have been repeatedly described as a robust in situ technique for predicting the bioavailability and toxicity of metals in water, soil and sediment [10,12,13,14,15]. Numerous studies have shown that it is superior in determining the distribution and bioavailability of one or more elements compared to conventional extraction methods [13,14,15].

The Zr oxide DGT sensors were used in this study to gain insight into the distribution of the labile forms of As, Cr, Mo, Sb, Se and V in the estuarine sediments of the Raša River. The elements Mo, Se, Cr and V are considered essential, although they can have toxic effects at elevated concentrations. As and Sb have also been the subject of scientific investigations for decades due to their toxic and carcinogenic effects. Accordingly, As, Cr, Sb, and Se have been listed as priority pollutants by the EPA (http://water.epa.gov accessed on 18 June 2021), and As compounds and Cr (VI) have been classified as Group 1 carcinogens by the International Agency for Research on Cancer (IARC; http://www.iarc.fr/ accessed on 20 June 2021).

In this study, the chemical composition of the water and sediments of the Raša River estuary was monitored in parallel with the distribution of DGT labile forms of As, Cr, Mo, Se, Sb and V along the sediment profiles with three main objectives: (1) to provide information on the overall water quality and the possible natural and anthropogenic sources of metals and metalloids in the estuary; (2) to gain knowledge on the lateral and vertical distribution of mobile forms of the oxyanions As, Cr, Mo, Se, Sb and V in the sediments of the Raša River estuary; (3) to determine the influence of the sediment on the chemical composition of the overlying water; and (4) to discuss the use of Zr oxide DGT sensors for depicting possible remobilization mechanisms of the mentioned oxyanions.

## 2. Results

### 2.1. Grain Size Distribution in Sediments

**Figure 1:**
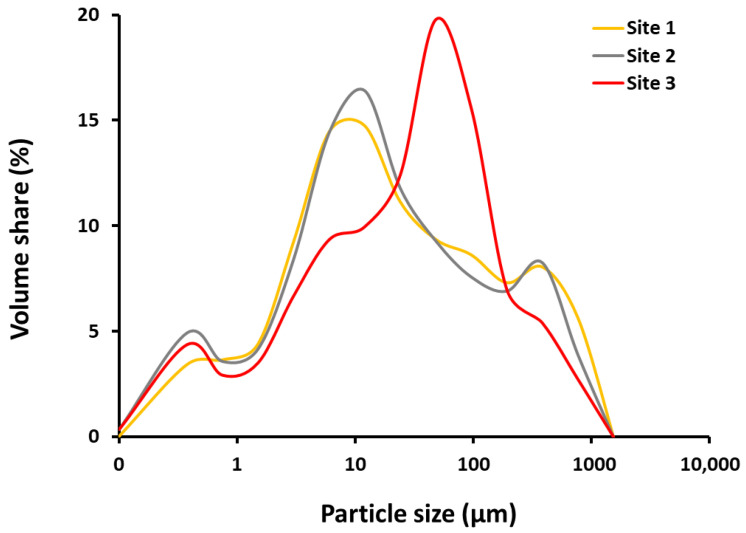
Grain size distribution in studied sediments.

Fine-grained particles predominate in the samples studied (Table 1), with average proportions of clay, silt and sand of 20%, 51% and 29%, respectively. In addition, all sediments were found to be very poorly sorted (So of 6.6–8.1) with bimodal to trimodal particle distribution (Figure 1).

### 2.2. Element Levels in Estuarine Water and Sediment

The results of the measurement of 25 elements (Al, As, Ba, Be, Cd, Co, Cr, Cs, Cu, Fe, Li, Mn, Mo, Ni, Pb, Rb, Sb, Se, Sn, Sr, Ti, Tl, U, V and Zn) in the estuarine water samples are presented in Table 2. Concentrations of Cd and Ni were below the detection limit of the analytical method (0.02 µg L^−1^). Water samples with salinity greater than 10 must be diluted prior to analysis to reduce dissolved solids to less than 2%; therefore, analysis of elements naturally present at very low concentrations (e.g., Cd and Ni) was not possible.

Mass fractions of 25 elements (Al, As, Ba, Be, Cd, Co, Cr, Cs, Cu, Fe, Li, Mn, Mo, Ni, Pb, Rb, Sb, Se, Sn, Sr, Ti, Tl, U, V and Zn) in studied sediments are presented in Table 3. 

### 2.3. Element Concentrations in Sediment Pore Water

**Figure 2:**
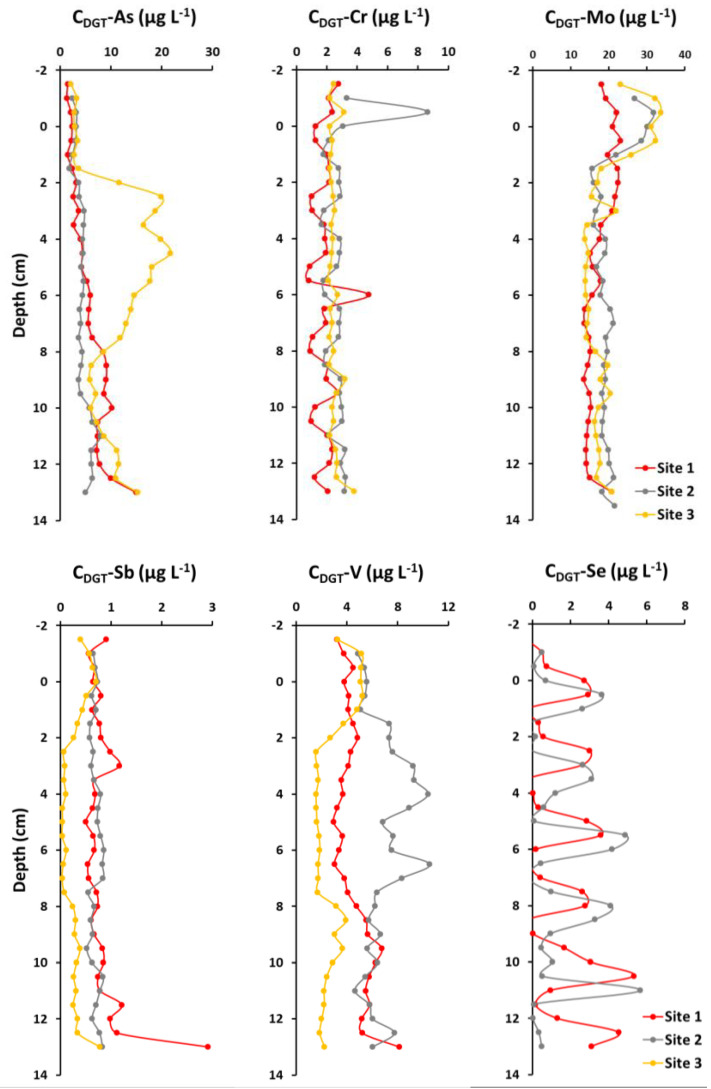
Vertical distributions of DGT-labile As, Cr, Mo, Sb, V and Se in the sediment-overlying water profiles at studied locations.

The distributions of DGT-labile oxyanions by depth are shown in Figure 2.

The C_DGT_-As showed almost no variability in the overlying water and an irregular variation in the sediment, which generally increased with depth and along the sampling profile, i.e., from site 1 to site 3. Among these, the Site 3 curve showed the greatest variability, reflected in a broad peak between 2 cm and 8 cm of the depth profile.

The variability trends of C_DGT_-Cr differed from those of the other oxyanions and exhibited the least overall variability, both with depth and along the sampling profile. However, the overlying water at Site 2 had the highest value at 1 cm (8.6 µg L^−1^), which was up to five times higher than in the other parts of the profile.

The C_DGT_-Mo curves showed an increase in surface water and a decrease in sediment down to 2 cm, followed by several small peaks and valleys in the rest of the area. In addition to the variations along the depth profiles, the C_DGT_-Mo curve showed an increase along the sampling profile, from site 1 to site 3.

In contrast, C_DGT_-Sb showed no differences between sampling sites, although the depth curves were characterized by a slight increase in overlying water followed by a slight decrease in sediment, and several non-coinciding minor peaks and curves along the depth profile.

C_DGT_-V behaves quite differently at depth than the other oxyanions, showing peaks at different depths in the sediments from the various sites, ranging from −2 cm to 2 cm (site 3), 2 cm to 5 cm and 6 cm to 8 cm (Site 2), and 8 cm to 11 cm (site 1), with the highest values generally occurring in the sediment from site 2.

Selenium is the only element whose C_DGT_-Se sediment was found below the detection limit at one site (Site 3), while values at the other two sites were scattered and showed no obvious trend.

## 3. Discussion

### 3.1. Granulometric Characteristics of Sediments 

The high proportion of fine-grained particles has been observed previously in the riverine and estuarine sediments of the region [4], and has been attributed to the intense weathering of Eocene flysch formations in the drainage area. When particles enter a stratified, saline, microtidal estuarine environment, rapid sedimentation occurs [19], reflected by the predominance of fine-grained sediments in the upper part and coarser sediments in the lower part of the estuary. This is consistent with an average grain size of 20 μm describing these sediments as coarse silts. 

The discrepancy in grain size distribution compared to the data reported by Vdović et al. [4] (22.8% clay, 66.6% silt, 10.6% sand) could be due to different sampling sites as well as lateral variability in estuarine sedimentation. According to Arbanas et al. [23], the average sedimentation rate in the estuary is 0.15 m year^−1^, which means that the sediments found were deposited recently, in the last one or two years.

### 3.2. Chemical Composition of Estuarine Water

In comparison with available literature values (Table 2), three groups of elements can be distinguished: (i) elements whose measured concentrations in the samples are lower than or comparable to previously published values for brackish water from the Raša River estuary (Be, Co, Cr, Sb, Se, Sn, Tl and V) [16]; (ii) conservative elements (Cs, Li, Mo, Rb, S, Sr and U) whose concentrations depend on salinity, so their values do not necessarily agree with previously published values due to differences in salinity of water at sampling sites; and (iii) elements present in significantly higher concentrations than those previously recorded for estuarine water in the wider area (Al, Ba, Fe, Mn, Pb, and Ti). Because the study area is characterised by karst springs that at times yield significant amounts of water and suspended sediment, the water samples collected may have contained elevated levels of suspended sediment. The 0.45-μm filtration process itself probably did not remove all particles, which is why we measured elevated concentrations of typical geogenic elements such as Al, Ba, Fe, Mn, and Ti in the water samples. Lead, a typical anthropogenic pollutant, was measured at the highest concentrations at site 3 compared to two other locations as well as Cu and Zn, indicating additional anthropogenic influences affecting water chemistry at the mouth of the Raša River. 

In order to clarify not only the origin but also the fate of individual elements in the observed estuary, element concentrations were monitored along the studied profile (Table 2). According to this, the elements can be divided into four groups: elements whose concentration increases along the profile (Be, Cs, Li, Mn, Mo, Rb, S, Sr, Tl, and U), elements whose concentration decreases along the profile (Al, Ba, Fe, Sn, and Ti), elements whose concentration first decreases and then increases significantly (Cu, Pb, and Zn), and elements whose concentrations are more uniform along the profile (As, Co, Cr, Sb, Se, and V). As mentioned above, the first group includes conservative elements, i.e., those whose concentrations in different parts of the estuary reflect the intensity of mixing of river and sea water. While various authors advocate both conservative and non-conservative behaviour of aluminium in estuaries and coastal areas [24], iron generally shows non-conservative removal and rapid decrease in the low salinity zone. Therefore, the decrease in the concentration of the second group of elements (Al, Ba, Fe, Sn, and Ti) along the profile may be attributed to removal due to colloidal flocculation upon contact of freshwater and seawater. The behaviour of the third group (Cu, Pb, and Zn), on the other hand, could probably be related to anthropogenic inputs (marina, harbour) highlighted by intense nautical activities, especially in summer [25].

It can be concluded that the chemical composition of estuarine water at the selected sites reflects the influence of the Raša River watershed enriched with suspended solids, the influence of seawater, which is inevitable in this disequilibrium estuary, as well as the influence of anthropogenic activities, which at least partially affect the content of certain elements.

### 3.3. Element Mass Fractions in Estuarine Sediments

The measured element mass fractions in the Raša Bay sediments were comparable to the element levels in the river sediments of the catchment [17]. The only exceptions were Co, Cr, Cu, Mn, Mo, Ni, Rb and Sr, which were found in higher levels compared to the river sediments of the catchment. Nevertheless, the contents of all these elements were within the range of values for estuarine sediments along the Adriatic coast and sediments of the Adriatic open sea [19,20,21]. However, in order to adequately assess the sediment quality at the studied sites, enrichment factors (EF) and geoaccumulation indices (I_geo_) were calculated (Table S1), using the upper values reported by Salminen et al. [17] for stream sediments in the region as a reference. 

The calculated EFs indicate moderate enrichment of sediments in Co, Cr, Mn, Mo, Ni, Sr, and Zn (at Site 3 only, Table S1). In contrast, the calculated geoaccumulation indices describe the sediments as uncontaminated to moderately contaminated with Co (at Site 1), and Cr and Ni (at Sites 2 and 3); and moderately contaminated with Mo (at Site 1), Ni (at Site 1), and Sr (Table S1). The apparent enrichment of the sediments with strontium is probably due to the fact that the analysis of the catchment sediments used a sediment fraction of 0.15 mm, where the Sr-rich carbonate component was removed during the sieving process, whereas the sediments in this study were sieved using a sieve with a pore size of 2 mm, which preserved the carbonate fractions.

It can be concluded that the chemical composition of the estuarine sediments at the selected sites primarily reflects the geological and hydrogeological background of the catchment, although they show slightly elevated levels of Co, Cr, Mo and Ni.

### 3.4. Sediment Pore Water Geochemistry

It is hypothesized that the observed variability in oxyanions along the depth profiles is because of several factors, namely ongoing early diagenetic processes in the sediments [26], disturbance of the sediments by benthic organisms [14], and the high-resolution sensitivity of the DGT instruments [15]. Decomposition of organic matter is the first stage of early diagenesis [26]. Because organic matter binds numerous trace elements (e.g., Cd, Cu, Cr, and Zn), subsequent decomposition in the sediment releases these elements back into the sediment pore water. However, the main pathway for mobilization of trace elements (e.g., As, Co, Cr, Fe, Mn, and V) in sediments is reductive dissolution of oxide and hydroxide minerals. Indeed, suboxic and anoxic conditions favor the release of elements bound to these phases due to organic matter degradation and associated oxygen consumption [26]. 

Because of the sensitivity of Fe and Mn oxides and hydroxides to changes in reducing conditions, their depth profiles in sediment pore water are a good indicator of the evolution of suboxic and anoxic conditions in the sediment [26,27]. In general, the release of manganese into the sediment pore water precedes the reduction of iron, Fe^3+^ to Fe^2+^, due to the reduction of Mn^4+^ to Mn^2+^ and is a good indicator of the transition from oxic to suboxic conditions in the sediment. Thus, elevated manganese concentrations in sediment pore water indicate the onset of suboxic conditions, while elevated iron concentrations in sediment pore water indicate the transition from suboxic to anoxic conditions [26,27]. Both events are usually accompanied by the release of elements bound to Mn- or Fe-oxyhydroxide [27,28,29]. Sometimes the coexistence of suboxic and anoxic conditions in sediments (e.g., during intense bioturbation) is reflected by a simultaneous increase in Mn and Fe concentrations, as well as elements bound to these phases, in sediment pore water. 

Although DGT devices only provide us with information about labile anions, their depth distribution can also provide some indication about redox conditions in the sediment. For example, the highest Cr concentrations in the sediment at Site 2 can most likely be associated with the local decomposition of organic matter, although Cr is usually linked with terrigenous detrital input [27,29]. The vertical profiles of Mo and As, on the other hand, are considered to indicate the onset of suboxic and anoxic conditions in the studied sediment. While manganese oxyhydroxides are regarded as the major sink for Mo in oxygenated conditions, the iron oxyhydroxides are the major carrier phase for As in the marine environment by transporting it through the water column and eventually to the pore water [27]. Upon dissolution of these phases, Mo and As are again released into the pore water [27,28,29]. Vanadium and antimony have also been associated with both iron and manganese oxide particles [27], while antimony, similar to chromium, has been associated with heavy fractions in sediments in some studies [29]. 

It can be concluded that suboxic conditions in the studied sediment start very early, already at the water-sediment interface, and are followed by the onset of anoxic conditions already at 2 cm depth. Differences in the vertical profiles of vanadium, an element that also readily precipitates with Fe-Mn -oxyhydroxides in sediments from different sites, also suggest lateral variability in the occurrence of individual conditions in the basin itself. However, for a more detailed insight into the diagenetic processes in this estuary, it is necessary to study a larger number of samples.

### 3.5. Potential Release Tendency of Metals and Metalloids from the Sediments

**Figure 3:**
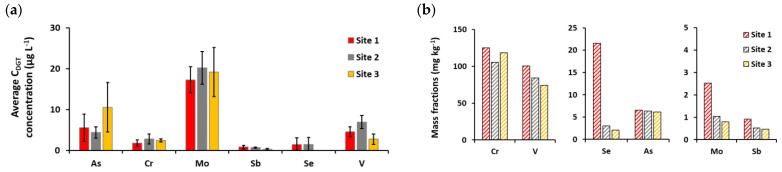
(**a**) Average C_DGT_ concentrations of As, Cr, Mo, Sb, Se and V (expressed in μg L^−1^); (**b**) Mass fractions of Cr, V, Se, As, Mo and Sb in sediments (expressed in mg kg^−1^) of the Raša estuary at studied locations.

The data obtained successfully demonstrate that the use of Zr oxide-DGT can provide additional information on the mobility of certain oxyanions in sediments. As can be seen in Figure 3a, despite the differences between sites, the highest concentrations in sediment pore water were observed for Mo at all three sites, up to 23.1 μg L^−1^ for all elements. Elevated Mo concentrations in sediment pore water are not as surprising, although As, Cr, and V are present in sediment in five to one hundred times higher mass fractions than Mo (Figure 3b), indicating that they likely originate from the dissolution of Mn oxides. Nevertheless, the element concentrations determined in sediment pore water were consistent with those previously published [26,29,30,31,32].

The diffusion fluxes of trace elements at the sediment-water interface were further calculated to provide information on whether the sediments act as a sink or a source for the elements. The diffusion coefficients and calculated values of diffusion fluxes for As, Cr, Mo, and Sb in sediment pore water are shown in Table 4. For As, Cr and Sb, the calculated diffusion fluxes were <1, which means that the studied sediments are sources of these elements. The average estimated fluxes expressed in μg m^−2^ day^−1^ are 1.0 for As, 0.04 for Cr, and 0.02 for Sb; and they contribute to 40% of As, 1.6% of Cr, and 2.9% of Sb of their average concentration in the bottom water of the Raša River estuary. The fluxes determined are comparable to those previously reported for marine and estuarine sediments [27,28,29] and suggest that sediments at sampling sites have a notable influence on the chemistry of the overlying water. 

## 4. Materials and Methods

### 4.1. Study Area

**Figure 4:**
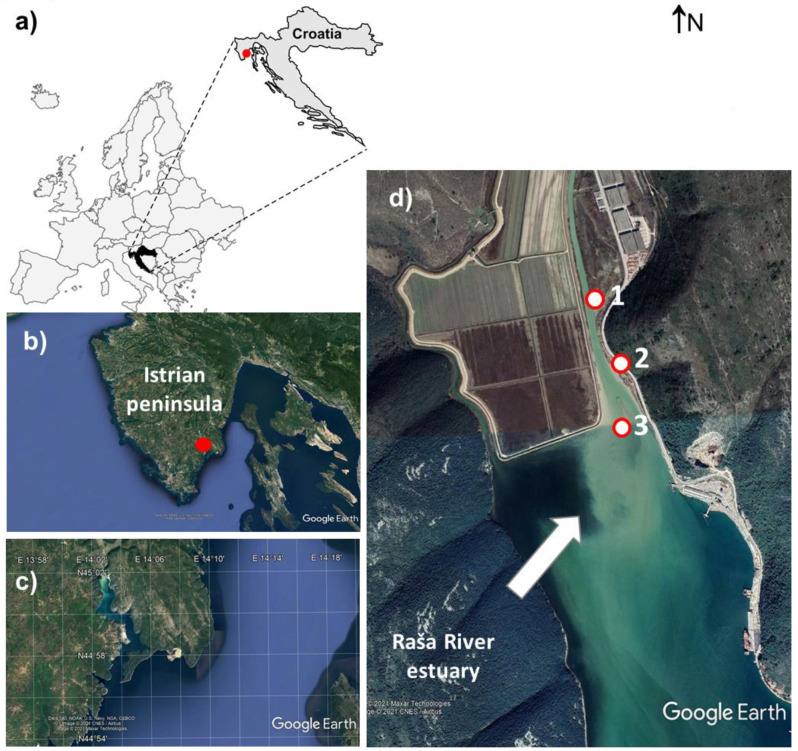
(**a**) Map of the study area, its geographical position; (**b**) Sampling area; (**c**) A more detailed view of the research area; (**d**) Studied estuary, with indicated sampling sites.

The study area includes the Bay of Raša (45°020 N 14°030 E) in the southeastern part of the Istrian peninsula, the westernmost part of Croatia (Figure 4). It is the lower part of the former valley of the Raša River, which was flooded by postglacial sea level rise. 

The region is characterized by thin to moderately thick red or brown clay loam soils of Chromic Cambisols and Chromic Luvisols classes [33] and a Mediterranean climate (mild, wet winters and hot, dry summers). Mesozoic sediment (mostly Cretaceous) and Paleogene (Paleocene, Eocene) rocks [34] with Raša coal seams [35] form the geological framework of the study area. Relatively high precipitation percolates over karstified limestone and feeds numerous watercourses that flow into the Raša River, which eventually flows into Raša Bay. Further details on the geology, hydrology and pedology of the wider area can be found elsewhere [34,35,36]. 

The Raša Bay represents a small, rock-bounded, microtidal, karstic estuary with low wave energy in the northeastern Adriatic, classified as a disequilibrium estuary. The Raša River is characterized by large fluctuations in water flow and variable loading of mineral particles. Most of this load is brought into the estuary in the form of fine-grained suspended sediment, consisting of carbonates and clay [4]. Sedimentation occurs at the salt wedge, resulting in a pro-grading estuarine delta. Salt-induced flocculation is the predominant process of sediment deposition.

Information on the geochemistry and mineralogy of Raša coal and the impact of its combustion products on the local soil, biota and water system is beyond the scope of this article and can be found elsewhere [2,3,35,36,37].

### 4.2. Sampling and Sample Preparation

The sediment and water samples were collected from the same sampling sites where the DGTs were employed. Details on GPS coordinates as well as the sampling depth is given in Table S2. Selected sites represent locations downstream of the coal washery from the time of Raša’s coal exploitation.

Surface water samples were collected in plastic bottles previously rinsed with sampling water. After sampling, water samples were filtered through 0.45 µm membrane (acetate) filter and acidified with nitric acid, 1% *suprapur* HNO_3_ (*v*/*v*). All samples were stored at 4 °C until further analysis. Prior to analysis samples were further diluted 20 times, acidified with 2% (*v*/*v*) HNO_3_ (65%, supra pur, Fluka, Steinheim, Switzerland) and In (1 µg L^−1^) as internal standard was added. 

Sediments were collected with sampling pipe by non-return valve and stored in a plastic bag. In the laboratory, all samples were air dried, sieved through a 2 mm sieve to remove the gravel fraction, and stored until further analysis.

For total element analysis, sediment sub-samples (0.05 g), previously homogenized in an agate mill, were subjected to a total digestion in the microwave oven (Multiwave 3000, Anton Paar, Graz, Austria) in a two-step procedure. The latter consisted of digestion with a mixture of 4 mL nitric acid (HNO_3_, 65%, pro analysi, Kemika, Zagreb, Croatia)-1 mL hydrochloric acid (HCl)-1 mL hydrofluoric acid (HF, 48%, pro analysi, Kemika, Zagreb, Croatia) followed by addition of 6 mL of boric acid (H_3_BO_3_, Fluka, Steinheim, Switzerland). Prior to analysis, digests were diluted tenfold, acidified with 2% (*v*/*v*) HNO_3_ (65%, supra pur, Fluka, Steinheim, Switzerland) and In (1 µg L^−1^) as internal standard was added. 

### 4.3. Analysis of Sediment Particle Size Distribution

The granulometric analysis of the sediments was carried out using a laser diffraction particle size analyzer (LS 13320, Beckman Coulter, Brea, CA, USA). Sample texture and statistical granulometric parameters were identified and calculated using the original Folk and Ward logarithmic graphical measures [38] in the GRADISTAT package [39]. Based on particle size distribution, sediment samples were classified according to Shepard [40]. 

### 4.4. DGT Preparation and Deployment

The Zr oxide DGT sensors were deployed in shallow muddy soil at a depth of approximately 30–40 cm. The average water temperature was 15.2 °C. However, the temperature of the sediment was 19.5 °C to 19.7 °C. Before inserting the DGT, a marker was placed on the side 3 cm below the top of the DGT window. Then the probes were carefully pushed into the sediment until the mark aligned with the sediment-water interface. After 24 h, the DGT probes were removed, rinsed with Milli-Q water, and taken to the laboratory, where water temperature was also recorded. 

In the laboratory, each resin gel was cut into 5 mm wide strips using a Teflon-coated blade. Each gel was placed in a centrifuge tube (10 mL), and 2 mL of a 0.2 M NaOH-0.5 M H_2_O_2_ mixed solution, previously stabilized at 4 °C, was added. The elution time was 4 h.

### 4.5. Multielement Analysis

Multielement analysis of the prepared digestions and extracts was performed by high-resolution inductively coupled plasma mass spectrometry (HR ICP- MS) using an Element 2 instrument (Thermo, Bremen, Germany). Typical instrument conditions and measurement parameters used throughout the work have been reported previously [41]. All samples were analyzed for total concentration of 25 elements (Al, As, Ba, Be, Bi, Cd, Co, Cr, Cs, Cu, Fe, Li, Mn, Mo, Ni, P, Pb, Rb, S, Sb, Sc, Se, Sn, Sr, Ti, Tl, U, V and Zn).

Quality control of the analytical procedure was performed by simultaneous analysis of the blank and the certified reference material for water (SLRS-4, NRC, Ottawa, ON, Canada), stream sediment (NCS DC 73309, China National Analysis Center for Iron and Steel, Beijing, China), and estuarine sediment (IAEA 405, IAEA, Vienna, Austria). For all elements, good agreement was obtained between the analyzed and certified concentrations within their analytical uncertainties (~10%). Additionally, measurement precision was determined through measurement repeatability. The latter was determined on the basis of six consecutive measurements of two samples, giving an average value of 3%.

### 4.6. Calculation of DGT Measured Concentration 

The mass of metal accumulated in the resin gel layer *M* (mg) over the deployment time is calculated using Equations (1) and (2):

(1) $$M=\frac{C_{e}\left(V_{1}+V_{2}\right)}{f_{e}}$$

(2) $$C_{DGT}=\frac{M\times \Delta g}{D\times A\times t}$$

where *C_e_* (mg L^−1^) is the concentration of the metal in the measured solution, *V*_1_ (L) is the volume of the binding gel, *V*_2_ (L) is the volume of the solution used for elution, and *f_e_* is the elution factor for each metal, *C_DGT_* (μg L^−1^) is the concentration of the metal measured by DGT, Δ*g* (cm) is the thickness of the diffusion layer, *D* (cm^2^ s^−1^) is the diffusion coefficient of the metal at 25 °C [13], *A* (cm^2^) is the exposure area, and *t* (s) is the deployment time.

To determine the extent to which the investigated sediments act as a source of oxyanions or their burial site, pore water diffusive fluxes were calculated for selected elements according to the first Fick’s law by assuming a linear concentration gradient between the sediment pore water at a depth of 10 cm and the bottom water:

(3) $$J_{i}\approx -\phi D_{}^{i}\left(\frac{\partial C_{i}}{\partial x}\right)$$

where *J_i_* (10^−6^ ng cm^−2^ s^−1^) is diffusive flux for element *i*, *φ* is sediment porosity, *D* (10^−6^ cm^2^ s^−1^) is diffusion coefficient, *δC_i_*/*δ_x_* is concentration gradient and *x* (cm) is depth.

Calculation of the diffusive fluxes was based on the diffusion coefficients (D, which were calculated using the Nernst-Einstein equation and reported by Li and Gregory [42], corrected for temperature (15 °C) and porosity (*φ*) using the mean porosity of estuarine sediments (*φ* = 0.807) according to Ullman and Aller [43]. The values of diffusive flux < 1 indicate diffusion in the direction of the bottom water, i.e., from the sediment into the water column. Analogously, values of diffusive flux > 1 indicate the retention of element of interest within the sediment. 

### 4.7. The Assessment of Sediment Pollution 

In order to quantify the levels of potential contamination of sediments with heavy metals, aluminium was used as the reference element [44]. For the background levels in the sediment, mean values of Al and other elements were taken (for the Istrian peninsula) from the Geochemical Atlas of Europe [17] for stream sediments.

The enrichment factors (EF) were calculated as follows:

(4) $$\mathrm{EF}=\frac{{\left(C_{n}/\;C_{Al}\;\right)}_{sample}}{{\left(B_{n}/\;B_{Al}\;\right)}_{background}}$$

where *C_n_* and *C_Al_* (sample) are the concentrations of an investigated element n and a reference element, respectively, in a sediment sample, while *B_n_* and *B_Al_* (background) are the concentrations of an investigated element n and a reference element, respectively, reported for the wider study area [17]. The contamination categories [45] are as follows: insufficient to minimal enrichment (EF = 2), moderate enrichment (EF = 2–5), considerable enrichment (EF = 5–20), very high enrichment (EF = 20–40), and extremely high enrichment (EF = 40).

The geoaccumulation *I_geo_* indices were calculated as follows:

(5) $$I_{geo}=log_{2}\frac{C_{n}}{1.5\times B_{n}}$$

where *C_n_* is the measured concentration of an element n in a soil sample, and *B_n_* is the geochemical background concentration of the same element in soil reported by FOREGS [17]. There are six classes of *I_geo_* [46]: practically uncontaminated (*I_geo_* ≤ 0), uncontaminated to moderately contaminated (0 < *I_geo_* < 1), moderately contaminated (1 < *I_geo_* < 2), moderately to heavily contaminated (2 < *I_geo_* < 3), heavily contaminated (3 < *I_geo_* < 4), heavily to extremely contaminated (4 < *I_geo_* < 5), and extremely contaminated (5 < *I_geo_*).

## 5. Conclusions

In Raša Bay, the chemical composition of estuarine water was found to be dominantly influenced by the catchment enriched in suspended sediments and freshwater-seawater mixing, and to a lesser extent by anthropogenic activities. The sediment composition, on the other hand, reflected the geological and hydrodynamic conditions in the drainage area and showed moderate enrichment of only several elements, Co, Cr, Mo and Ni. The reason for this is most likely a combination of natural and anthropogenic factors in the catchment and, although described as moderate, should not be ignored. 

The geochemical composition of the water and sediment at studied locations does not reflect the impact of past mining activities; however, the release of certain elements (e.g., Mo, As, Cr) from the sediment suggests that the sediment of this estuary may be a source of certain elements. Even at very low concentrations, their transfer into sediment pore water and further into the water column can have adverse effects on the biota in the estuary. 

In dynamic systems such as the Raša estuary, which are subject to rapid sedimentation and where reducing conditions already prevail in the subsurface sediment, it is important to constantly monitor not only the total content of elements in the water and sediments, but also their mobile and consequently bioavailable forms.

The study clearly shows that bioavailable forms of elements should be considered when assessing the environmental quality of coastal systems, as the absence of such information may lead to incomplete assessment, especially in dynamic coastal systems such as estuaries. In this regard, DGTs are extremely useful as simple, rapid and inexpensive tools for assessing the mobility of oxyanions in order to evaluate the state of the environment of a given coastal system. 

## Figures and Tables

**Table 1:** Granulometric characteristics of sediment samples.

(%)	Site 1	Site 2	Site 3
clay	20.8	21.5	17.8
silt	49.6	51.9	51.5
sand	29.6	26.6	30.7

**Table 2:** Comparison of mean element concentrations in the surface layer of the Raša River estuary with literature values, expressed in μg L^−1^ or * mg L^−1^.

Element	Raša River Estuary (This Study)			Raša River Estuary [16]	Fonte Gaja (Natural Freshwater Spring) [16]	Stream Water of the Drainage Area [17]	Karin Sea [18]	Velebit Channel (Surface) [18]	Velebit Channel (Bottom) [18]
	Site 1	Site 2	Site 3						
Al	31.3	29.2	13.1	6.5	3–37	2.10–4.10	4.94	6.15	10.2
As	0.62	0.51	0.61		0.40–1.40	0.18–0.30	0.82	1.17	2.50
Ba	40.2	37.0	16.5	31.3	14.6	10.1–14.9	10.5	11.3	7.84
Be	0.06	0.06	0.11	0.08	0.007	<0.009			
Cd				0.21	0.03–0.2	0.016–0.022			
Co	0.05	0.04	0.03	0.06	0.04	0.11–0.16	0.38	0.21	0.040
Cr	0.40	0.28	0.26	0.60	0.5–5	0.28–0.38	0.72	0.58	1.18
Cs	0.06	0.07	0.10	0.04	0.004	0.002–0.003	0.12	0.13	0.320
Cu	0.55	0.33	1.81	0.60	0.43–10	0.88–1.20	0.83	1.16	1.72
Fe	33.0	29.8	12.6	2.1	1–390	30–67	5.52	4.71	10.0
Li	45.4	50.5	81.4	27.5	0.46	0.70–2.10	68.4	75.2	175
Mn	8.68	8.84	9.49	2.2	0.5–25	3.90–6.70	0.26	0.44	3.34
Mo	3.91	4.26	6.06	33.1	2.04	0.22–0.37	4.07	4.78	12.2
Ni				1.30	0.35–20	3.93–6.37	2.20	2.98	4.04
Pb	0.49	0.39	2.12	0.20	0.09–1	0.093–0.160			
Rb	28.7	31.7	51.0	19.5	1.04	0.69–0.94	48.5	54.3	127
S *	219	243	396	153	5100				
Sb	0.09	0.07	0.07	0.37	0.07	0.02–0.04			
Se	0.48	0.34	0.37	3.50	1.09	0.13–0.19			
Sn	0.34	0.17	0.12	0.35	0.35				
Sr	2316	2508	3691	1797	191	50–190	3337	3691	8415
Ti	3.43	1.01	0.34	0.10	0.37	0.3–0.4			
Tl	0.012	0.012	0.014	0.04	0.01	0.003–0.005			
U	1.27	1.32	1.77	10.8	0.75	0.32–0.63	1.68	1.72	3.62
V	1.02	1.05	0.77	1.70	1.37	0.16–0.32	1.58	1.74	1.80
Zn	4.15	2.17	5.04	4.20	2.5–267	2.68–4.00			

Salinity values reported for water in the Karin Sea, Velebit channel (surface) and Velebit channel (bottom) were 14, 17 and 38, respectively [18].

**Table 3:** Comparison of element concentrations in sediments of the Raša River estuary with literature values, expressed in mg kg^−1^ or * g kg^−1^.

Element	Raša River Estuary (This Study)			Stream Sediments of the Drainage Area [17]	Zrmanja River Estuary [19]	Northern and Central Adriatic [20]	Rijeka Harbour [21]	Adriatic [22]
	Site 1	Site 2	Site 3					
Al *	43.8	34.3	35.4	44.5–54.5	2.1–5.2	52.4	1.77	
As	6.52	6.37	6.12	6–9	5.66–16.7		21.4	19.7
Ba	168	148	156	118–205	98.7–186		86.3	
Be	1.17	0.96	0.91	0.78–1.00	0.693–1.81		0.92	
Cd	0.44	0.32	0.29	0.40–0.51	0.32–0.41	0.11	1.07	
Co	13.8	11.2	11.1	6.0–8.0	4.55–9.86	9.9	12.9	
Cr	125	106	119	50–63	34.4–98.2	85.8	71.6	57.3
Cs	5.74	3.98	3.89	5–6	2.84–7.32		1.77	
Cu	32.0	22.4	22.0	13–22	6.11–18.4	8.0	145	
Fe *	24.7	20.2	19.0	25.0–31.1	1.06–2.68	23.8	2.74	0.9
Li	46.2	35.3	34.7	30.5–39.3	26.8–61.4		38.9	
Mn	507	504	511	310–387	170–351	542	343	130
Mo	2.53	1.04	0.79	0.63–0.81	0.64–2.27		3.53	
Ni	72.3	53.6	54.6	15.0–21.0	12.2–45.4	37.9	86.1	23.8
Pb	18.4	17.7	18.5	17.0–20.5	18–33.8	29.7	227	52.7
Rb	93.7	68.3	67.2	57.6–70.0	39.7–96.4		31.3	34.3
Sb	0.91	0.53	0.47	1.15–1.62	0.40–0.89		1.62	
Se	0.48	0.34	0.37				1.05	
Sn	0.34	0.17	0.12	1.80–2.25	1.56–2.94		6.66	
Sr	313	317	312	71.0–99.0	136–247		213	
Ti *	3.0	2.5	2.6	3.1–3.7	1.5–3.3	2.4		0.995
Tl	0.46	0.34	0.33	0.48–0.59	0.36–0.79		0.39	
U	2.57	2.91	1.97	1.0–3.0	1.57–2.62		2.61	
V	101	84.2	74.2	62–78	37.1–97	53.1	72.4	
Zn	92.9	66.2	95.8	45–71	76.6–112	76.2	369	206

**Table 4:** Diffusion coefficients and derived values of average diffusive fluxes for sediment pore water at studied locations.

Element	Diffusion Coefficient (D/10^−6^ cm^2^ s^−1^)	Diffusive Flux (J/10^−6^ ng cm^−2^ s^−1^)		
		Site 1	Site 2	Site 3
As	6.3	−2.4	−2.1	−2.4
Cr	7.8	0.08	0.3	−0.08
Mo	6.9	3.4	6.5	8.2
Sb	5.8	−0.05	−0.01	0.21

## Data Availability

Not available.

## Supplementary Materials

The following are available online, Table S1 Calculated enrichment factors (EF) and geoaccumulation indices (I_geo_) for the sediments studied; Table S2 Longitude and latitude data for the sampling locations, including the depth of the water column at those locations.

## Samples Availability

Samples of the compounds are not available from the authors.
