# Supplementary material for: Evaluation of the Potential Release Tendency of Metals and Metalloids from the Estuarine Sediments: Case Study of Raša Bay
Source: Molecules. 2021 Nov 3;26(21):6656. doi: 10.3390/molecules26216656 (PMC8587412; doi:10.3390/molecules26216656)
Supplement: Supplementary file 1 [file molecules-26-06656-s001.zip › molecules-1414366-supplementary.pdf]

Table S1. Calculated enrichment factors (EF) and geoaccumulation indices (I_{geo}) for the sediments studied. Values marked in bold indicate moderate enrichment (EF = 2-5) or describe the sediments as uncontaminated to moderately contaminated (I_{geo} = 0-1) or moderately contaminated (I_{geo} = 1-2). Values marked in red indicate considerable enrichment (EF = 2-5) of the sediments.

	EF			Igeo		
	**Site 1**	**Site 2**	**Site 3**	**Site 1**	**Site 2**	**Site 3**
**Al**	1.0	1.0	1.0	-0.9	-1.3	-1.2
**As**	0.9	1.1	1.0	-1.1	-1.1	-1.1
**Ba**	1.0	1.1	1.2	-0.9	-1.1	-1.0
**Be**	1.5	1.5	1.4	-0.4	-0.6	-0.7
**Cd**	1.1	1.0	0.9	-0.8	-1.3	-1.4
**Co**	**2.1**	**2.2**	**2.1**	**0.2**	-0.1	-0.1
**Cr**	**2.5**	**2.7**	**2.9**	**0.4**	**0.2**	**0.3**
**Cs**	1.2	1.1	1.0	-0.6	-1.2	-1.2
**Cu**	1.8	1.6	1.5	0.0	-0.6	-0.6
**Fe**	1.0	1.0	0.9	-0.9	-1.2	-1.3
**Li**	1.5	1.4	1.4	-0.4	-0.7	-0.8
**Mn**	1.6	**2.1**	**2.0**	-0.2	-0.2	-0.2
**Mo**	**3.9**	**2.0**	1.5	**1.1**	-0.2	-0.6
**Ni**	**4.3**	**4.1**	**4.0**	**1.2**	**0.8**	**0.8**
**Pb**	1.1	1.4	1.4	-0.7	-0.8	-0.7
**Rb**	1.7	1.6	1.5	-0.2	-0.6	-0.6
**Sb**	0.7	0.5	0.4	-1.4	-2.2	-2.4
**Se**	-	-	-	-	-	-
**Sn**	0.2	0.1	0.1	-3.3	-4.3	-4.8
**Sr**	**3.9**	5.1	**4.9**	**1.1**	**1.1**	**1.1**
**Ti**	1.0	1.1	1.1	-0.9	-1.1	-1.1
**Tl**	1.0	0.9	0.9	-0.9	-1.4	-1.4
**U**	1.1	1.5	1.0	-0.8	-0.6	-1.2
**V**	1.6	1.7	1.5	-0.2	-0.5	-0.7
**Zn**	1.6	1.5	**2.1**	-0.2	-0.7	-0.2

Table S2. Longitude and latitude data for the sampling locations, including the depth of the water column at those locations.

	**Longitude**	**Latitude**	**Depth**
**Site 1**	45° 2' 11.7'' N	14° 2' 47.76'' E	30 cm
**Site 2**	45° 2' 0.18'' N	14° 2' 50.28'' E	40 cm
**Site 3**	45° 1' 51.54'' N	14° 2' 48.18'' E	35 cm
